# A staphylococcal cyclophilin carries a single domain and unfolds via the formation of an intermediate that preserves cyclosporin A binding activity

**DOI:** 10.1371/journal.pone.0210771

**Published:** 2019-03-29

**Authors:** Soham Seal, Soumitra Polley, Subrata Sau

**Affiliations:** Department of Biochemistry, Bose Institute, Kolkata, West Bengal, India; Jamia Millia Islamia, INDIA

## Abstract

Cyclophilin (Cyp), a peptidyl-prolyl *cis*-*trans* isomerase (PPIase), acts as a virulence factor in many bacteria including *Staphylococcus aureus*. The enzymatic activity of Cyp is inhibited by cyclosporin A (CsA), an immunosuppressive drug. To precisely determine the unfolding mechanism and the domain structure of Cyp, we have investigated a chimeric *S*. *aureus* Cyp (rCyp) using various probes. Our limited proteolysis and the consequent analysis of the proteolytic fragments indicate that rCyp is composed of one domain with a short flexible tail at the C-terminal end. We also show that the urea-induced unfolding of both rCyp and rCyp-CsA is completely reversible and proceeds via the synthesis of at least one stable intermediate. Both the secondary structure and the tertiary structure of each intermediate appears very similar to those of the corresponding native protein. Conversely, the hydrophobic surface areas of the intermediates are comparatively less. Further analyses reveal no loss of CsA binding activity in rCyp intermediate. The thermodynamic stability of rCyp was also significantly increased in the presence of CsA, recommending that this protein could be employed to screen new CsA derivatives in the future.

## Introduction

The cyclophilins (EC: 5.2.1.8) represent a family of highly conserved peptidyl-prolyl *cis/trans* isomerase (PPIase) enzymes those are expressed by most living organisms, and some giant viruses [1–5]. These proteins control protein folding by catalyzing the *trans* to *cis* isomerization of the peptidyl bonds those precede proline residues. These enzymes also influence numerous other cellular processes including protein trafficking, transcription, cell differentiation, apoptosis, protein secretion, T-cell activation, and signal transduction. In addition, these folding catalysts play critical roles in developing cardiovascular diseases, rheumatoid arthritis, viral infections, cancer, diabetes, sepsis, asthma, aging, neurodegenerative diseases, and microbial infections [2, 3, 6–11]. The catalytic activities of the cyclophilins are typically inhibited by cyclosporin A (CsA), a cyclic peptide harboring eleven amino acid residues [1]. A ternary complex, formed by the association of CsA-cyclophilin complex with calcineurin, prevents the dephosphorylation of the transcription factor NF-AT that, in turn, blocks the expression of cytokines from T-lymphocytes [12–14]. The reduction of T-cell activity by CsA has made it extremely useful in clinics, particularly for preventing graft rejection after organ and bone marrow transplantation [2]. However, the severe side effects of CsA have restricted its use [15] and promoted to develop many CsA analogs with no immunosuppressive activity [2, 10, 11, 16–18]. Some of these CsA analogs though yielded promising results have not been approved yet.

Cyclophilins, located in the cytosol and membrane or cell organelles, are composed of either single domain or multiple domains [1–3, 19]. Usually, one domain in the multidomain cyclophilins, like the single domain cyclophilins, is employed for both the catalysis of prolyl isomerization and CsA binding, whereas their rest domains are involved in the variety of functions including controlling of PPIase activity [19]. The PPIase domains of cyclophilins have a β-barrel conformation that is constituted with eight anti-parallel β-strands, two-three α-helices, and several connecting loops [1, 20, 21]. Their hydrophobic active sites are made using the side-chains of amino acid residues from most of the β-strands and loops. While thirteen residues are required for binding CsA [1, 20– 22], eleven residues in the active site are involved in the binding of a tetrapeptide substrate [1, 23]. Of the residues, nine residues are used by both CsA and substrate for binding.

The linear polypeptide chains synthesized in the living cells become functional only when these molecules are folded into proper three-dimensional forms. To experimentally understand the mechanism of protein folding, native/denatured forms of proteins are gradually unfolded/refolded followed by monitoring their conformational changes using a suitable probe [24, 25]. The unfolding/refolding study usually indicates whether the folding of a protein occurs by a two-state or by a multi-state mechanism through the generation of no or multiple intermediates. Additionally, unfolding studies intimate about the stability of proteins in the presence of ligands or mutations [26–32]. Furthermore, such studies have greatly influenced many biotechnological fields including drug discovery [33–39]. Cyclophilins belonging to the same class or different class harbor a structurally conserved PPIase domain [1–3, 19]. However, these enzymes are not identical at the amino acid sequence level, indicating that their unfolding mechanism may be different. Thus far, unfolding mechanisms of only a few cyclophilins [40–43] were studied though these proteins were considered as the promising drug targets [2, 3, 10, 11].

*Staphylococcus aureus*, a pathogenic bacterium, harbors a gene that encodes a putative cyclophilin (SaCyp) of 197 amino acid residues [42]. A gene chip-based investigation has demonstrated that SaCyp (UniProt Code: A0A0H3K705) is not induced by stress [44]. Conversely, a gene silencing-based study has indicated that this protein is not required for the growth of *S*. *aureus* [45]. On the other hand, computational analyses have revealed that SaCyp shares significant sequence homology with the PPIase domains of cyclophilins from different living organisms including human [42, 46]. The putative PPIase domain formed by nearly the entire region of SaCyp also carries a CsA binding site [42]. Various experimental studies have collectively suggested that SaCyp carries PPIase activity, exits as a monomer, binds CsA, and is involved in the *S*. *aureus*-mediated hemolysis and virulence [42, 46]. Specifically, nuclease, an *S*. *aureus*-encoded virulence factor seems to be folded by SaCyp [46]. Interestingly, a point mutation in SaCyp that abolished its PPIase activity partly refolded a denatured nuclease [47]. An *S*. *aureus* mutant carrying the above mutation also exhibited pathogenicity in mouse and possessed hemolytic activity [47]. Jointly, SaCyp could be exploited in developing and screening the novel antistaphylococcal agents capable of preventing the infections caused by the multidrug-resistant *S*. *aureus* strains, a global concern today [48–50]. As the gene expressing SaCyp is not essential [45, 47], *S*. *aureus* will less likely develop resistance to the newly designed or screened SaCyp inhibitors. Additionally, new CsA analogs, discovered using the structural or unfolding data of SaCyp, may also be helpful for treating other diseases. Recently, a study has indicated that the drug-bound or drug-unbound form of SaCyp unfolds via the generation of one intermediate in the presence of guanidine hydrochloride (GdnCl) [42]. In addition, there was a significant stabilization of SaCyp in the presence of CsA [42]. Proteins are sometimes denatured by a different pathway in the presence of different unfolding agent [51–54]. Thus far, the unfolding mechanism and stability of SaCyp have not been verified using other denaturants. The structural and functional properties of GdnCl-made SaCyp/SaCyp-CsA intermediate are also currently not known with certainty. Moreover, the predicted single domain structure of SaCyp [42] has not been confirmed by any biochemical study. Herein, we have studied the domain structure and urea-induced unfolding of a recombinant *S*. *aureus* Cyp (rCyP) [42] using various probes. Our limited proteolysis data indicate that rCyp is a single-domain protein with a flexible tail at its C-terminal end. The urea-induced equilibrium unfolding of both rCyp and rCyp-CsA occurred via the synthesis of at least one stable intermediate. Interestingly, each intermediate has a native protein-like structure. Of the intermediates, rCyp intermediate has nearly full CsA binding activity.

## Materials and methods

### Materials

Many materials including acrylamide, anti-his antibody, alkaline phosphatase-goat anti-mouse antibody, ANS (8-anilino-1-naphthalene sulfonate), bis-acrylamide, chymotrypsin, CsA, Phenylmethane sulfonyl fluoride (PMSF), isopropyl β ᴅ-1-thiogalactopyranoside (IPTG), proteinase K, protein marker, trypsin, and urea were used in the present study.

### Construction and purification of a recombinant SaCyp

To obtain clues about the domain structure and the unfolding mechanism of SaCyp, a recombinant SaCyp (designated rCyp) was used in the present study. To construct rCyp, a 593 bp DNA fragment, amplified using an *S*. *aureus* genomic DNA and the primers 824–1 and 824–2, was cloned to plasmid pET28a as described [42, 55]. A pET28a derivative harboring no mutation in the cloned fragment was picked up and named p1350. The *S*. *aureus*-specific DNA insert in p1350 encodes rCyp that is composed of the entire SaCyp plus a polyhistidine tag attached to its N-terminal end.

rCyp was purified from the p1350 carrying *E*. *coli* BL21 (DE3) cells using a standard method [42]. Briefly, an exponentially grown culture of the above cells was exposed to 200 μM of IPTG for 4 h at 37°C. The induced cells collected after centrifugation were successively washed with 0.9% NaCl, resuspended in buffer A [20 mM Tris-HCl (pH 8.0), 20 mM imidazole, 10 μg/ml PMSF, 5% glycerol, and 500 mM NaCl], and lysed by sonication. The cell supernatant prepared by removing the debris from the broken cells was mixed with a one-tenth volume of Ni-NTA agarose solution. After a brief incubation for 5 min at 4°C, the mixture was loaded on a purification column followed by the draining out of buffer A by gravity flow. The column was washed with a modified buffer A that had 40 mM imidazole instead of 20 mM imidazole. Finally, rCyp was eluted from the column using another modified buffer A that contained 200 mM imidazole. The purified protein was dialyzed against buffer B [20 mM Tris-HCl (pH 8.0), 5% glycerol, and 300 mM NaCl] for 14–16 h at 4°C prior to performing any experiment.

### Basic protein techniques

Many regularly-used protein methods [55–57] were exploited in the current investigation for specific purposes. The content of rCyp in buffer B was determined by a standard procedure as stated [56, 57]. In brief, 10 μl of rCyp was mixed with 1 ml of Bradford solution carrying Coomassie Brilliant Blue G250, methanol, and phosphoric acid. Similarly, different amounts (0–20 μg) of BSA were added to different tubes carrying an identical volume of Bradford solution. After incubation for 5 min at room temperature, the OD595 values of the solutions were determined followed by their plotting against the corresponding BSA concentrations. The amount of rCyp was estimated from the equation of the resulting straight line. The theoretical mass of monomeric rCyp was determined by analyzing its sequence (see below) with ProtParam (web.expasy.org), a computational tool. The molar concentration of rCyp was estimated using both its theoretical mass and content in buffer B. To produce rCyp-CsA, we incubated 20–50 μM CsA with 10–25 μM rCyp in buffer B for 30 min at 4°C [42].

To check the purity of rCyp or the generation of rCyp fragments, we have performed sodium dodecyl sulfate (SDS)-polyacrylamide gel electrophoresis (PAGE) as reported [55–57]. In short, a resolving gel was prepared first by adding 4 ml of 30% acrylamide-bis-acrylamide solution, 3 ml of resolving buffer [1.5 M Tris-Cl (pH 8.8) and 0.4% SDS], 90 μl of 10% ammonium persulfate, 12.5 μl of TEMED and 1.8 ml of double distilled water. A stacking gel, made by assembling 333 μl of 30% acrylamide-bis-acrylamide solution, 665 μl of stacking buffer [0.5 M Tris-Cl (pH 6.8) and 0.4% SDS], 20 μl of 10% ammonium persulfate, 3 μl of TEMED and 985 μl of double distilled water, was poured on the solidified resolving gel. A comb was inserted to generate wells. After loading the protein samples on the wells of the set gel, electrophoresis was performed for 2–3 h at 80 V using a running buffer [25 mM Tris-Cl (pH 8.3), 250 mM Glycine and 0.1% SDS].

The protein bands in the SDS-polyacrylamide gel were visualized by a standard method as described [56, 57]. Briefly, the polyacrylamide gel collected after electrophoresis was incubated for 2–12 h at room temperature in a newly-made staining solution [0.25% Coomassie Brilliant Blue R250, 45% methanol, and 10% acetic acid]. The staining solution was replaced with a destaining solution [20% methanol, and 10% acetic acid] and the incubation was continued to remove the stain.

To detect the presence of a polyhistidine tag in rCyp and variants, we have performed Western blot analysis by a standard procedure as demonstrated [56, 57], In a nutshell, proteins from the polyacrylamide gels were transferred to the PVDF membrane followed by its sequential incubation with 3% BSA, mouse anti-his antibody, and alkaline phosphatase-tagged goat anti-mouse antibody for 1–2 h at room temperature. The membrane was washed twice with TBST buffer [50 mM Tris-Cl (pH 7.5), 150 mM NaCl, and 0.1% Tween 20] and once with TBS buffer [TBST carrying no detergent] after each incubation. Lastly, the protein bands in the membrane were detected using a chromogenic solution made with nitro blue tetrazolium chloride and 5-bromo-4-chloro-3-indolyl-phosphate.

### Functional investigation

The PPIase activity of rCyp was evaluated by RNase T1 (ribonuclease T1) refolding assay as reported [29, 42]. In sum, 16 μM RNase T1 in buffer B was incubated with 5.6 M GdnCl for 12–16 h at 10°C. The refolding of denaturated RNase T1 was started at 10°C by diluting it eighty fold with the buffer B carrying rCyp (120 nM) or no rCyp. The refolding rate of RNase T1 was determined by recording its tryptophan fluorescence (λ_ex_ and λ_em_ = 295 nm and 323 nm) using a Hitachi F-3000 spectrofluorometer having the band-pass of 2.5 nm for excitation and 5 nm for emission. The enzymatic activity, *k*_cat_/*K*_m_, was estimated by analyzing the fluorescence data with the following equation:

$$k_{\mathrm{cat}}/K_{m}=(k_{p}-k_{a})/[E]$$ (1)

where [E], *k*_p_ and *k*_a_ denote the concentration of rCyp, the first-order rate constant in the presence of rCyp, and the first-order rate constant in the absence of rCyp, respectively. The first-order rate constant (*k* = *k*_p_ or *k*_a_) was estimated by nonlinear fitting of the fluorescence data to the ‘one phase association’ equation from GraphPad Prism (GraphPad Software Inc.).

Previously, our modeling study indicated that one *S*. *aureus* Cyp molecule binds to one molecule of CsA [42]. Considering similar interaction between rCyp and CsA, the related equilibrium dissociation constant (*K*_d_) was estimated by a standard method [42] with minor modifications. Briefly, the intrinsic Trp fluorescence spectra (*λ*_ex_ = 295 nm and *λ*_em_ = 300–400 nm) of rCyp (2 μM) in the presence of varying concentrations (0–4 μM) of CsA were recorded using a fluorescence spectrophotometer. The fluorescence intensity values (at *λ*_max_ = 343 nm), extracted from the spectra, were rectified by deducting the related buffer fluorescence and by adjusting for volume changes. Lastly, the *K*_d_ value was estimated by fitting the fluorescence data to a standard equation (Eq 2) using GraphPad Prism (GraphPad Software Inc.).

$$Y=(B_{\max}[X])/(K_{d}+[X])$$ (2)

where [*X*], *Y*, and *B*_max_ represent the concentration of CsA, the amount of fluorescence change at any concentration of CsA, and the maximum fluorescence change upon saturation of rCyp with CsA, respectively.

### Limited proteolysis

To know whether rCyp carries any domain, limited proteolysis of this protein was separately executed by different proteolytic enzymes using standard methods [57, 58]. Briefly, a buffer B [42] solution carrying rCyp (10 μM) and an enzyme (0.025–0.2 μM) was incubated at ambient temperature. At different time points, an aliquot (50 μl) was pulled out and mixed with an SDS gel loading dye [55]. All of the aliquots were boiled prior to their resolution by a Tris-glycine SDS-13.5% PAGE. After staining with Coomassie brilliant blue, the photograph of acrylamide gel was captured as stated [57].

To determine the molecular masses of rCyp fragments, a MALDI-TOF analysis (Bruker Daltonics, Germany) was performed mostly as stated earlier [58]. Briefly, rCyp was exposed to a proteolytic enzyme for 10–20 min followed by the termination of reaction using PMSF at a final concentration of 0.5 mM. To inactivate the enzyme, the reaction mixture was incubated with benzamidine sepharose for 30 min. The supernatant collected after centrifugation was dialyzed against a 20 mM NH_4_HCO_3_ containing buffer for 4 h at 4°C. Finally, the supernatant obtained after centrifugation of the dialyzed sample was mixed with an equal volume of sinapinic acid. After drying the mixture on a sample plate, it was analyzed by MALDI-TOF mass spectrometry. The yielded m/z spectra were used to calculate the molecular masses of the rCyp fragments using the standard equations as reported [59].

### Spectroscopic observation

To know about the different structural elements of rCyp and rCyp-CsA in buffer B [42], the ANS fluorescence (λ_ex_ and λ_em_ = 360 nm and 400–600 nm), intrinsic tryptophan (Trp) fluorescence (λ_ex_ and λ_em_ = 295 nm and 300–400 nm), near-UV circular dichroism (CD) (250–320 nm), and far-UV CD (200–260 nm) spectra of these proteins were recorded at room temperature by the methods mostly as described before [42, 51, 57]. We used 25 μM protein for the near-UV CD spectroscopy and 10 μM protein for the far-UV CD or the fluorescence spectroscopy. The path length of the cuvette in the near-UV CD spectroscopy was 5 mm, whereas that in the far-UV CD spectroscopy was 1 mm. During the recording of the Trp fluorescence spectra, the band passes for excitation and emission were kept 2.5 nm and 5 nm, respectively. The ANS concentration used in the study was 100 μM. The fluorescence or CD intensity values were rectified by subtracting the reading of buffer from the reading of the same buffer carrying protein.

### Unfolding and refolding of proteins

To study the unfolding pathway of rCyp and rCyp-CsA, these proteins (10 μM each) were exposed to varying concentrations (0–8 M) of urea for ~18 h at 4°C as stated [51, 57]. Protein aliquots were always treated with the freshly prepared urea solution. To understand the effects of denaturant on the different structures of proteins, the ANS fluorescence, intrinsic Trp fluorescence, and the CD spectra of the urea-treated/untreated proteins were recorded as described above. The spectroscopic signals were corrected by deducting the reading of urea containing buffer from the reading of the same buffer carrying protein.

To check whether the proteins denatured by 7–8 M urea can refold upon removal of urea, they were dialyzed against buffer B [42] for 12–16 h at 4°C prior to the recording of their Trp fluorescence spectra as described above. The spectra of equal extent of both native and unfolded proteins were also recorded for comparison. To see whether the refolded rCyp is functional, we performed RNase T1 refolding as stated above.

### Transverse urea gradient gel electrophoresis

The unfolding of rCyp and rCyp-CsA were also monitored by a standard transverse urea gradient gel electrophoresis (TUGE) with minor modifications [29, 51, 60]. Briefly, a gel having a 10–7% acrylamide gradient and a 0–8 M urea gradient was made using a 10% acrylamide solution and a 7% acrylamide solution containing 8 M urea. Both the acrylamide solutions were prepared using an alkaline buffer [500 mM Tris-Cl (pH 8.5)]. At 0 and 8 M urea, the concentrations of acrylamide were 10% and 7%, respectively. After turning the solidified gel 90°, protein (60 μg) in an SDS-less loading buffer [55] was loaded on its generated well. The gel electrophoresis was carried out for 4–6 h at 65 V using an appropriate running buffer [25 mM Tris-Cl (pH 8.5) and 250 mM glycine] in the cold room (4°C). The staining of the gel was performed as stated above.

### Analysis of unfolding data

To gather clues about the unfolding pathways and the stabilities of rCyp and rCyp-CsA, the unfolding curves, produced using their spectroscopic and TUGE data, were fit to either the two-state (N ↔ U) equation (Eq 3) or the three-state (N ↔I↔U) equation (Eq 4) using GraphPad Prism as described [24, 25, 29, 57].

$$Y=[Y_{N}+Y_{U}\exp(-(\Delta G^{W}-m[C])/RT]/[1+\exp(-(\Delta G^{W}-m[C])/RT]$$ (3)

$$Y=\{Y_{N}+Y_{I}\exp(-(\Delta G^{W1}-m1[C])/RT+Y_{U}\exp(-((\Delta G^{W1}-m1[C])+(\Delta G^{W2}-m2[C]))/RT\}/\{1+\exp(-(\Delta G^{W1}-m1[C])/RT+exp(-((\Delta G^{W1}-m1[C])+(\Delta G^{W2}-m2[C]))/RT\}$$ (4)

where *Y*, *Y*_N_, *Y*_I_, *Y*_U_, *R*, and *T* denote the observed spectroscopic signal or mobility of the protein at any urea concentration, the spectroscopic signal or mobility of the protein in the completely folded state, the spectroscopic signal of the protein for the N ↔ I unfolding transition, the spectroscopic signal or mobility of the protein in the completely unfolded state, universal gas constant, and absolute temperature in Kelvin, respectively. Conversely, *m*, *m*_1_, and *m*_2_ indicate the cooperativity parameters for the N ↔ U, N ↔ I, and I ↔ U unfolding transitions. On the other hand, Δ*G*^W^, Δ*G*^W1^, and Δ*G*^W2^ represent the free energy changes for the N ↔ U, N ↔ I, and I ↔ U transitions. The urea concentrations at the midpoint of N ↔ U transition (*C*_m_), N ↔ I transition (*C*_m1_), and the I ↔ U transition (*C*_m2_) were obtained by diving Δ*G*^W^, Δ*G*^W1^, and Δ*G*^W2^ with *m*, *m*_1_, and *m*_2_, respectively.

The difference of free energy change between rCyp-CsA and rCyp for the N ↔ U transition (ΔΔ*G*), N ↔ I transition (ΔΔG*1*), and the I ↔ U transition (ΔΔ*G2)* were determined using the Eqs 5, 6 and 7 as reported [24].

$$\Delta \Delta G=<m>\Delta C_{m}$$ (5)

$$\Delta \Delta G1=<m_{1}>\Delta C_{m1}$$ (6)

$$\Delta \Delta G2=<m_{2}>\Delta C_{m2}$$ (7)

where ‹*m*›, ‹*m*_*1*_›, and ‹*m*_*2*_› indicate the average values of *m*, *m*_1_, and *m*_*2*_ derived from the N ↔ U, N ↔ I, and I ↔ U unfolding processes of rCyp-CsA and rCyp, respectively. Conversely, Δ*C*_m_, Δ*C*_m1_, and Δ*C*_m2_ denote the difference in the *C*_m_, *C*_m1_, and *C*_m2_ values estimated from the N ↔ U, I ↔ U, and I ↔ U unfolding processes of rCyp-CsA and rCyp.

The fraction of unfolded rCyp molecules (*f*_U_) was determined from the CD or Trp fluorescence data using the following equation [29]:

$$f_{U}=(X_{N}-X)/(X_{N}-X_{U})$$ (8)

where *X*_N_, *X*_U_, and X represent the spectroscopic signal of rCyp in the fully folded state, the spectroscopic signal of rCyp in the completely unfolded state, and the spectroscopic signal of rCyp at any urea concentration, respectively. The values of *X*_N_ and *X*_U_ were calculated from the straight lines developed using the spectroscopic signals of rCyp at very low and very high urea concentrations.

## Results

### Domain structure of rCyp

**Fig 1:**
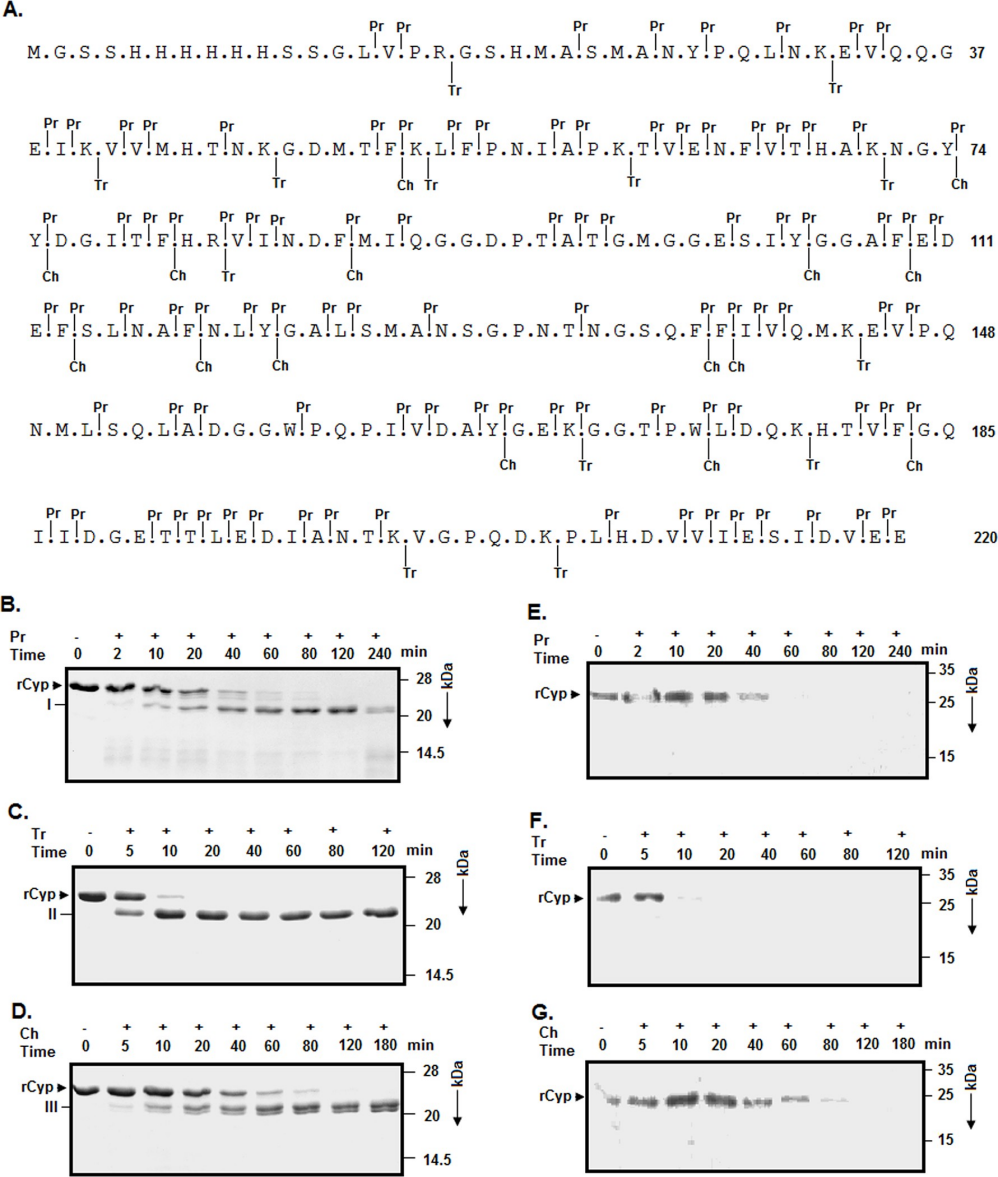
Characterization of rCyP by limited proteolysis. (A) The amino acid sequence of rCyp with the cut sites of chymotrypsin (Ch), trypsin (Tr), and proteinase K (Pr). The predicted cleavage sites, found out by analyzing the sequence of rCyp with PeptideCutter (web.expasy.org/peptide_cutter), are shown at the top and bottom of the sequence using vertical lines. The polyhistidine tag of rCyp is composed of residues 1–23. The rCyp fragments, generated from the partial digestion of rCyp with the either of Pr (B), Tr (C), and Ch (D), were separated by different SDS-13.5% PAGE. Arrowhead and I-III represent the intact rCyp and the major rCyp fragments, respectively. (E, F, G) Western blotting experiment. The Pr-, Tr-, and Ch-generated rCyp fragments were analyzed using an anti-his antibody. We have mentioned the mass of each marker protein at the right-hand sides of the blot and gel images.

A modeling study previously indicated that the cyclophilin, encoded by *S*. *aureus*, could be a single domain protein [42]. To confirm this proposition, we have individually performed limited proteolysis [51, 57–59, 61] of rCyp with trypsin, chymotrypsin, and proteinase K. Each of these enzymes is computationally determined to have higher than ten cleavage sites, which are distributed along the entire sequence of rCyp (Fig 1A). This protein will mostly remain insensitive to the above enzymes if it is really composed of only one domain. We have noted the generation of primarily one proteolytic fragment from rCyp at the initial stage of its cleavage with proteinase K (Fig 1B). One major fragment was also made at the early period of digestion of rCyp with trypsin (Fig 1C) or chymotrypsin (Fig 1D). The proteinase K-, trypsin- and chymotrypsin-generated fragments are designated as fragment I, fragment II and fragment III, respectively. All of the fragments remained stable during the entire period of digestion. The intensities of the fragments were gradually increased with the increase of time of digestion. Their molecular masses are about ~2–3 kDa less than that of rCyp, indicating that its digestion occurred differently by a different enzyme. While fragment I seemed to be produced by the cleavage at one or both ends of rCyp, fragment II was possibly originated due to the removal of any one of its end. On the other hand, fragment III might have been generated in an unconventional way. The peptide bonds formed by Phe, Tyr, and Trp residues are usually cleaved by chymotrypsin with high efficiency, whereas those are made with Leu, Met, and His residues are digested by this enzyme with less efficiency (web.expasy.org/peptide_cutter). All of the higher sensitive cut sites of chymotrypsin are located in the rCyp region that is made by its residues 52 to 183 (Fig 1A). As the removal of last 36 residues or the first 52 residues of rCyp by chymotrypsin would contribute to the mass loss of 4 kDa or more, fragment III might have been generated due to the cleavage at the less chymotrypsin-sensitive bonds at its ends. Our Western blot analyses show no interaction between the proteolytic fragments and anti-his antibody (Fig 1E–1G), indicating the loss of polyhistidine tag from the N-terminal end of rCyp in the presence of the above enzymes.

To find out the cut sites in rCyp, the masses of the above proteolytic fragments (I-III) were estimated using MALDI-TOF mass spectrometry as described [58]. The m/z spectrum shows that there was a generation of two major peaks from rCyp digested with proteinase K (S1A Fig). Conversely, trypsin (S1B Fig)- or chymotrypsin (S1C Fig)-digested rCyp resulted in largely one major peak as expected. As the two peaks obtained from the proteinase K-digested rCyp were fused with each other, the fragment I might be composed of two proteolytic fragments (designated as Ia and Ib) having a little difference in molecular mass. The single major peak originated from the trypsin-digested rCyp most possibly corresponds to fragment II. Similarly, the peak yielded from the chymotrypsin-cleaved rCyp might be due to fragment III. The molecular masses of the above rCyp fragments, calculated using the m/z spectral data (S1 Fig), were found to vary from 21493.63 to 22050.73 Da (Table 1). Using the predicted cut site data of rCyp (Fig 1A), different proteolytic fragments were generated followed by the determination of their masses using a computational tool (web.expasy.org/protparam). The rCyp fragments whose theoretical masses (Table 1) nearly matched with the experimental masses of fragments Ia, Ib, II, and III are composed of the amino acid residues Ser 23 to Val 218, Ser 23 to Glu 219, Gly 18 to Glu 220, and Ala 22 to Glu 220, respectively (Fig 1A). Thus, five peptide bonds, made by the rCyp residues Arg 17 and Gly 18, Met 21 and Ala 22, Ala 22 and Ser 23, Val 218 and Glu 219, and Glu 219 and Glu 220, showed sensitivity to the proteolytic enzymes employed in the investigation. Of the susceptible peptide bonds, three bonds are in the polyhistidine tag carrying region of rCyp and the rest bonds are in the extreme C-terminal end of this enzyme (Fig 1A). Collectively, both ends of rCyp might be exposed to its surface.

**Table 1:** Mass and composition of the proteolytic fragments.

Enzyme	rCyp fragments	Experimental mass of rCyp fragments^A^ (Da)	Theoretical mass of rCyp fragments^B^ (Da)	Composion of rCyp fragments^C^
Proteinase K	Ia	21493.63	21448.06	Ser23-Val218
Proteinase K	Ib	21564.43	21577.16	Ser23-Glu219
Trypsin	II	22050.73	22189.81	Gly18-Glu220
Chymotrypsin	III	21973.69	21777.35	Ala22-Glu220

^A^The masses of the rCyp fragments were estimated using MALDI-TOF data (S1 Fig).

^B^The theoretical masses of fragments with the designated residue carrying regions

^C^, calculated by ProtParam (web.expasy.org/protparam), are very close to those determined using MALDI-TOF data.

### Unfolding of proteins

**Fig 2:**
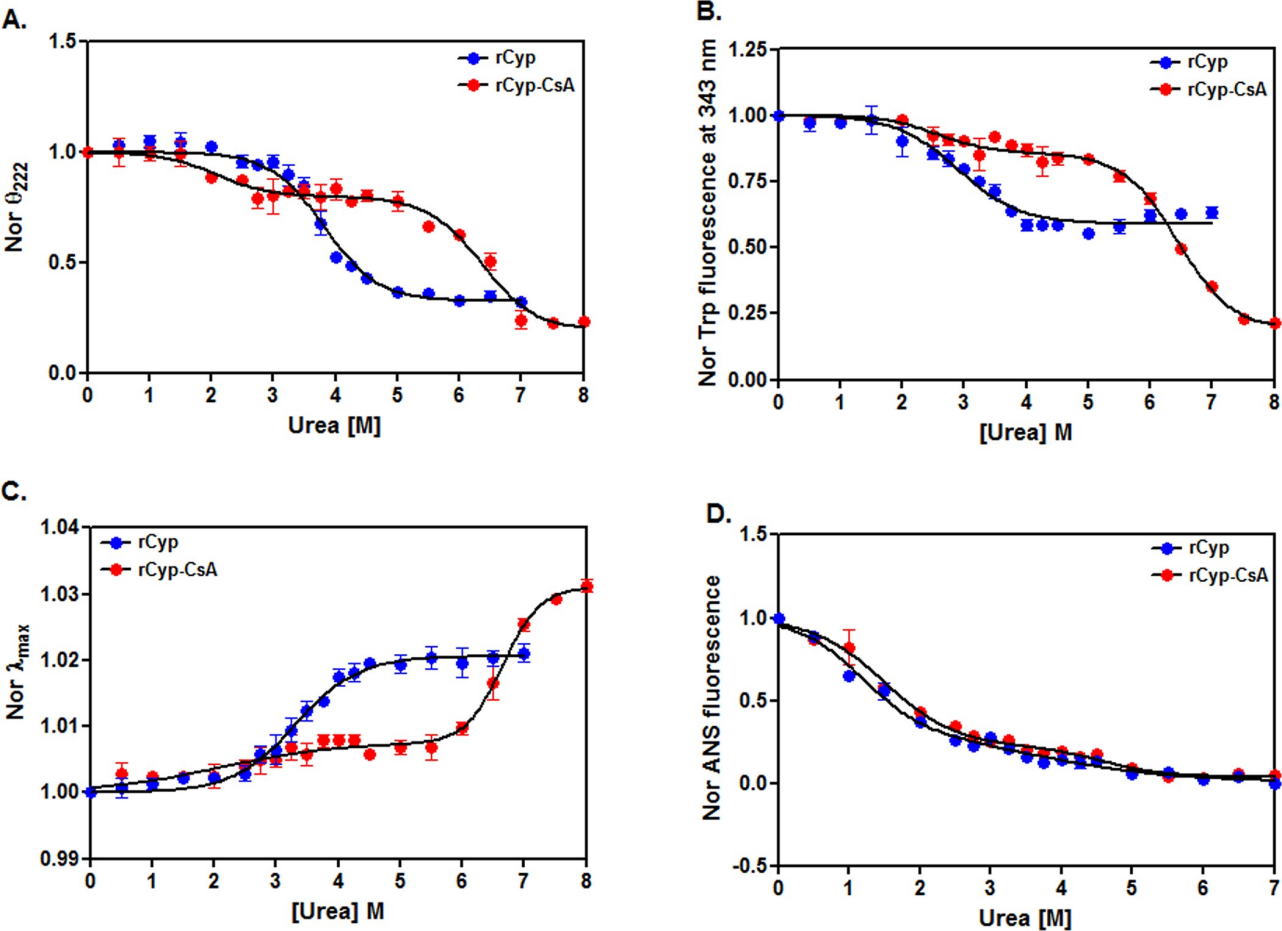
Unfolding studied by spectroscopic tools. (A) The ellipticity values of rCyp and rCyp-CsA at 222 nm, extracted from their far-UV CD spectra (S2 Fig), were normalized and plotted against the corresponding urea concentrations as described [51]. (B) The intrinsic Trp fluorescence intensity values of rCyp (at 343 nm) and rCyp-CsA (at 341 nm), obtained from the respective spectra (S2 Fig), were normalized and plotted as above. (C) The λ_max_ values of rCyp and rCyp-CsA, derived from the Trp fluorescence spectra (S2 Fig), were similarly plotted. (D) The ANS fluorescence intensity values of rCyp, and rCyp-CsA at 480 nm, collected from the related spectra (S2 Fig), were identically normalized and plotted. All lines through the spectroscopic signals denote the best-fit lines.

The unfolding pathways of many proteins (e.g. glucose oxidase, human placental cystatin, hexokinase, FKBP22, and trigger factor) appeared dissimilar in the presence of different denaturants including urea and GdnCl [51–54, 62]. Previously, both rCyp and rCyp-CsA in the presence of GdnCl were unfolded via the production of one intermediate [42]. To check whether the urea-induced unfolding of these proteins would follow the similar pathway, their far-UV CD, intrinsic Trp fluorescence, and ANS fluorescence spectra were separately recorded in the presence of 0 to 7/8 M urea (S2 Fig) A monophasic curve is obtained for rCyp when the ellipticity values at 222 nm were plotted against the matching urea concentrations. Conversely, such a curve generated for rCyp-CsA was biphasic in nature (Fig 2A). A monophasic curve for rCyp and a biphasic curve for rCyp-CsA were also obtained when we plotted their Trp fluorescence intensity (Fig 2B) or the associated *λ*_max_ (Fig 2C) values against the related urea concentrations. The *λ*_max_ values of both proteins were shifted to 350 nm when there was a saturation of fluorescence intensity. All of the biphasic curves show the transitions at ~1.5/2–2.75/3 M and ~5/5.5–7/7.5 M urea, respectively. Unlike the curves obtained using the CD and Trp fluorescence data, the curves, prepared using the ANS fluorescence intensity values of rCyp and rCyp-CsA, look very similar and possibly carry two transitions (Fig 2D).

**Fig 3:**
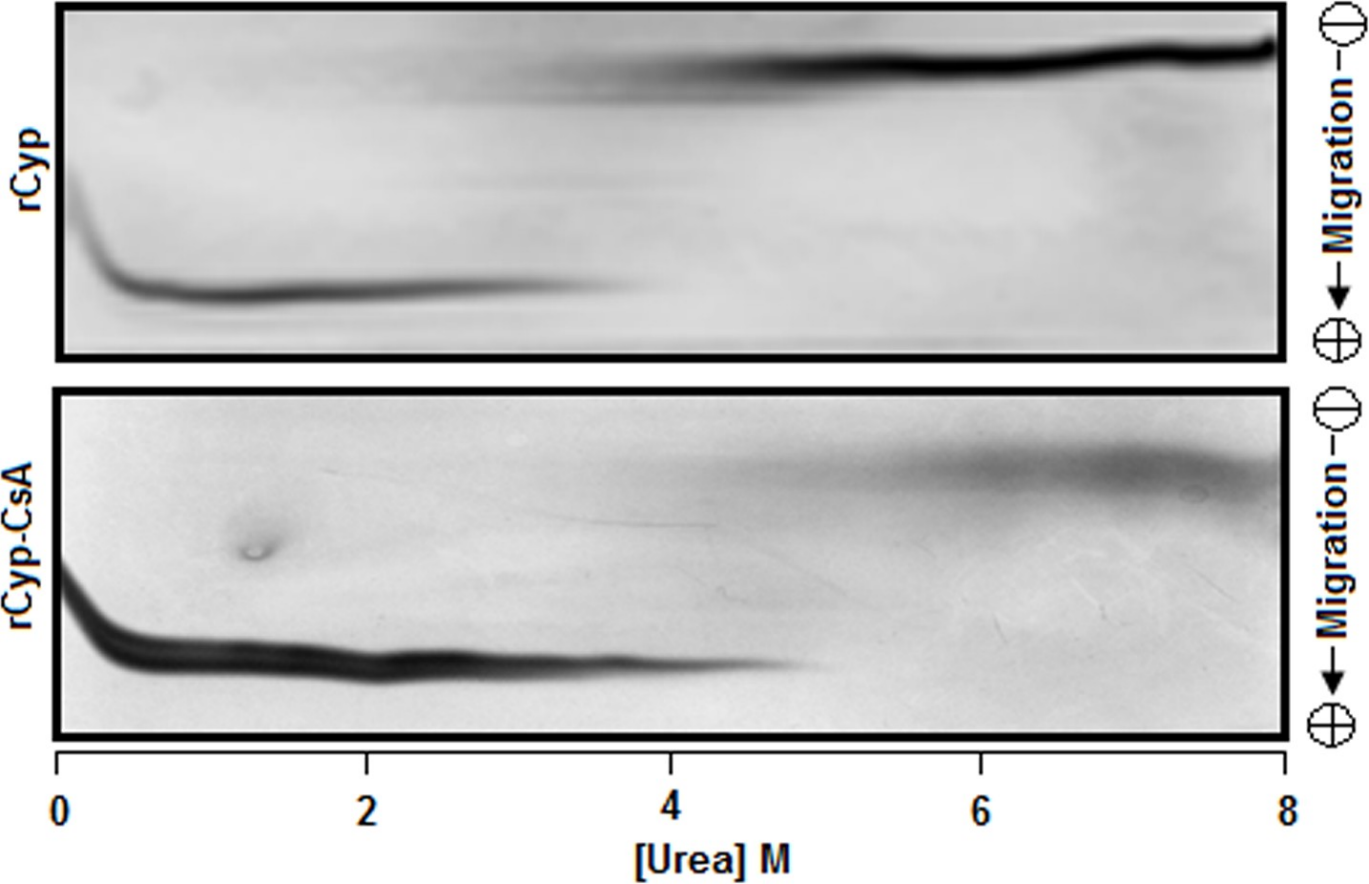
Transverse urea gradient polyacrylamide gel electrophoresis of proteins. Both rCyp and rCyp-CsA were separately analyzed by the Transverse urea gradient polyacrylamide gel electrophoresis as described [51, 58].

To verify the above unfolding data, we have also investigated the unfolding of rCyp and rCyp-CsA using transverse urea gradient gel electrophoresis [51], a biochemical probe. The migration of rCyp or rCyp-CsA across the urea gradient gel yielded an S-shaped protein band having nearly a clear transition region (Fig 3). The rCyp-specific protein band shows a transition at ~3.25–4.25 M urea, whereas, that of rCyp-CsA results in a transition region at ~4.75–5.75 M urea, indicating that the initiation of the unfolding of drug-bound rCyp occurred at higher urea concentration. The faded transition region also suggests a slow unfolding reaction [58, 60].

**Fig 4:**
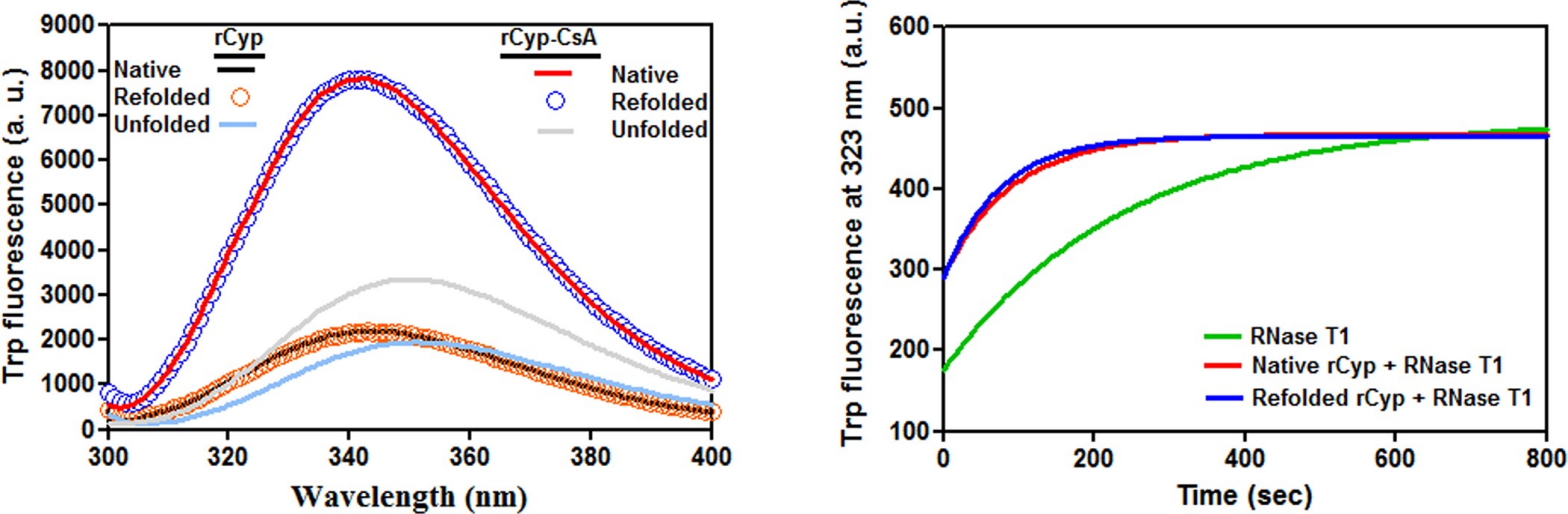
Refolding of the urea-exposed proteins. (A) The intrinsic Trp fluorescence spectra of the unfolded, native, and refolded rCyp or rCyp-CsA. (B) RNase T1 activity of refolded and native rCyp.

The reversibility of the unfolding reaction was checked by recording the Trp fluorescence spectra of the native, denatured, and the probable refolded forms of rCyp and rCyp-CsA as described [42]. We have observed that the Trp fluorescence spectra of the native protein and the related refolded protein have completely coincided with each other (Fig 4A). Additional RNase T1 refolding assay reveals that there is nearly a complete restoration of the PPIase activity in the renatured rCyp (Fig 4B). In sum, both rCyp and rCyp-CsA were unfolded by a reversible pathway in the presence of urea.

### Unfolding mechanism of the protein

**Fig 5:**
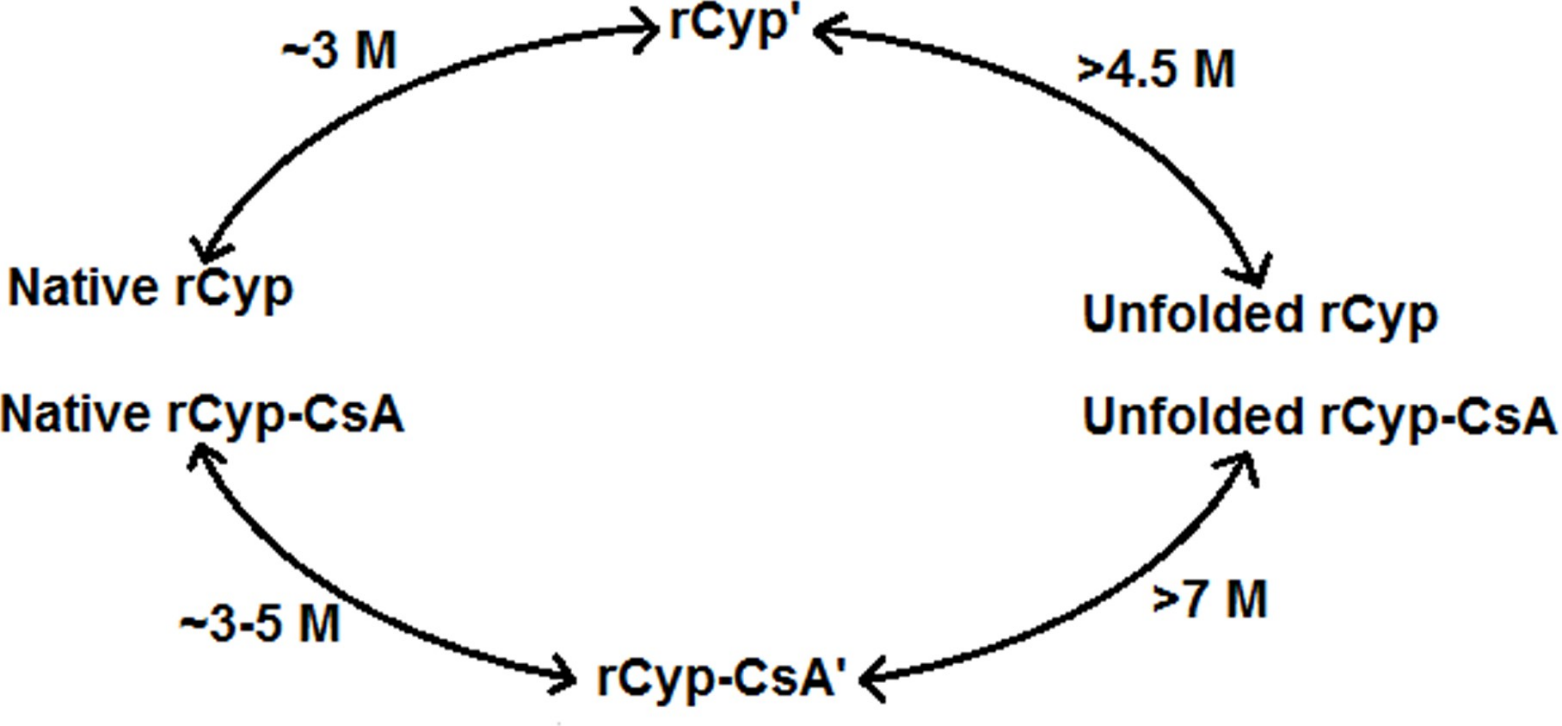
A graphic presentation of the urea-induced unfolding of rCyp and rCyp-CsA. The unfolding intermediates are denoted by rCyp’ and rCyp-CsA’.

To accurately determine the mechanism of the urea-induced unfolding of rCyp and rCyp-CsA, all of the unfolding curves were examined using different models [24, 25, 57]. Each rCyp-specific curve, generated using CD or Trp fluorescence signals, exhibited the best fitting with a two-state model [24]. The *C*_m_ values, obtained from the fitted CD (Fig 2A), Trp fluorescence intensity (Fig 2B) and the *λ*_max_ (Fig 2C) data of rCyp, are 3.82±0.04 M, 2.91 ± 0.07 M, and 3.30±0.06 M urea, respectively. Conversely, the rCyp-specific curve, produced using ANS fluorescence signals, fit best to the three-state model [25] with the resulted *C*_m1_ and *C*_m2_ values of ~1.18 M and ~3.68 M urea (Table 2), respectively. Thus, the ANS fluorescence data suggest the formation of a rCyp intermediate at ~3 M urea (Fig 5). Two additional pieces of evidence have supported the above proposal. The fractions of denatured rCyp molecules, estimated from both the CD (Fig 2A) and Trp fluorescence data (Fig 2B), were plotted against 0–7 M urea and the resulted curves did not coincide with each other (S3A Fig). The non-overlapping of such curves indicates the formation of unfolding intermediate [25]. Secondly, the phase diagram [29, 63], used to know about the formation of the hidden unfolding intermediate of proteins, was developed by plotting the Trp fluorescence intensities of rCyp at 320 nm against its fluorescence intensities at 365 nm (S3B Fig). The yielded non-linear plot again suggests the formation of rCyp intermediate(s) at 0–7 M urea.

**Table 2:** Thermodynamic parameters of protein unfolding.

Protein	Assay method	Fitted equation	*C*_m_ / *C*_m1_ / *C*_m2_ (M)	*m / m*_*1*_*/ m*_*2*_ (kcal mol^-1^M^-1^)	Δ*G*^W^ / Δ*G*^W1^ / Δ*G*^W2^(kcal M^-1^)	ΔΔ*G* / ΔΔ*G1* / ΔΔ*G*2 (kcal M^-1^)
rCyp	ANS fluorescence	Three-state	1.18±0.03 3.68±0.19	1.69±0.01 0.74±0.02	1.99±0.03 2.74±0.07	
	TUGE	Two-state	3.79±0.36	1.81±0.21	6.83±0.15	
rCyp-CsA	ANS fluorescence	Three-state	1.51±0.02 4.61±0.21	1.33±0.03 1.70±0.19	2.01±0.12 7.82±0.53	0.55±0.10 1.12±0.08
	TUGE	Two-state	5.53±0.56	1.44±0.19	7.92±0.27	2.81±0.02

The thermodynamic parameters were estimated from ANS fluorescence (Fig 2D) and TUGE (Fig 3) data using the Eqs 3–7 [24, 25].

All of the unfolding data of rCyp-CsA (Fig 2), accumulated from our spectroscopic studies, fit best to a three-state model, clearly suggesting the formation of a rCyp-CsA intermediate in the presence of urea. While the *C*_m1_ and *C*_m2_ values yielded from the CD data of rCyp-CsA are 2.09±0.22 M and 6.37±0.09 M, those from its Trp fluorescence data are 2.58±0.17 and 6.5±0.05 M urea. Conversely, the *C*_m1_ and *C*_m2_ values estimated from the ANS fluorescence signals of rCyp-CsA are ~1.51 M and ~4.61 M urea, respectively (Table 2). Jointly, a rCyp-CsA intermediate might have been generated at ~3–5 M urea (Fig 5).

### Stability of the protein

A protein is usually stabilized when it binds a ligand [28, 29, 33, 36, 37, 39, 42]. To see whether the stability of rCyp is increased in the presence of CsA, the values of different thermodynamic parameters (Table 2), obtained from the ANS fluorescence data of rCyp and rCyp-CsA (Fig 2D), were further analyzed as stated above. The data show that the *C*_m1_ and *C*_m2_ values of rCyp-CsA are significantly higher than those of rCyp (all *p* values <0.05). The difference of free energy change between rCyp and rCyp-CsA (i.e. ΔΔ*G1* or ΔΔ*G2*) is more than ~0.5 kcal M^-1^ (Table 2). The thermodynamic parameters, determined by fitting the TUGE data (Fig 3) with the two-state equation [24], are also presented in Table 2. The yielded Δ*G*^W^ and *C*_m_ values of rCyp-CsA were noted to be significantly higher than those of rCyp (all *p* values ≈ 0.03). The free energy change ΔΔ*G* between rCyp and rCyp-CsA is about 2.8 kcal M^-1^ (Table 2). Taken together, we suggest that the stability of rCyp is increased in the presence of CsA.

### Properties of unfolding intermediates

**Fig 6:**
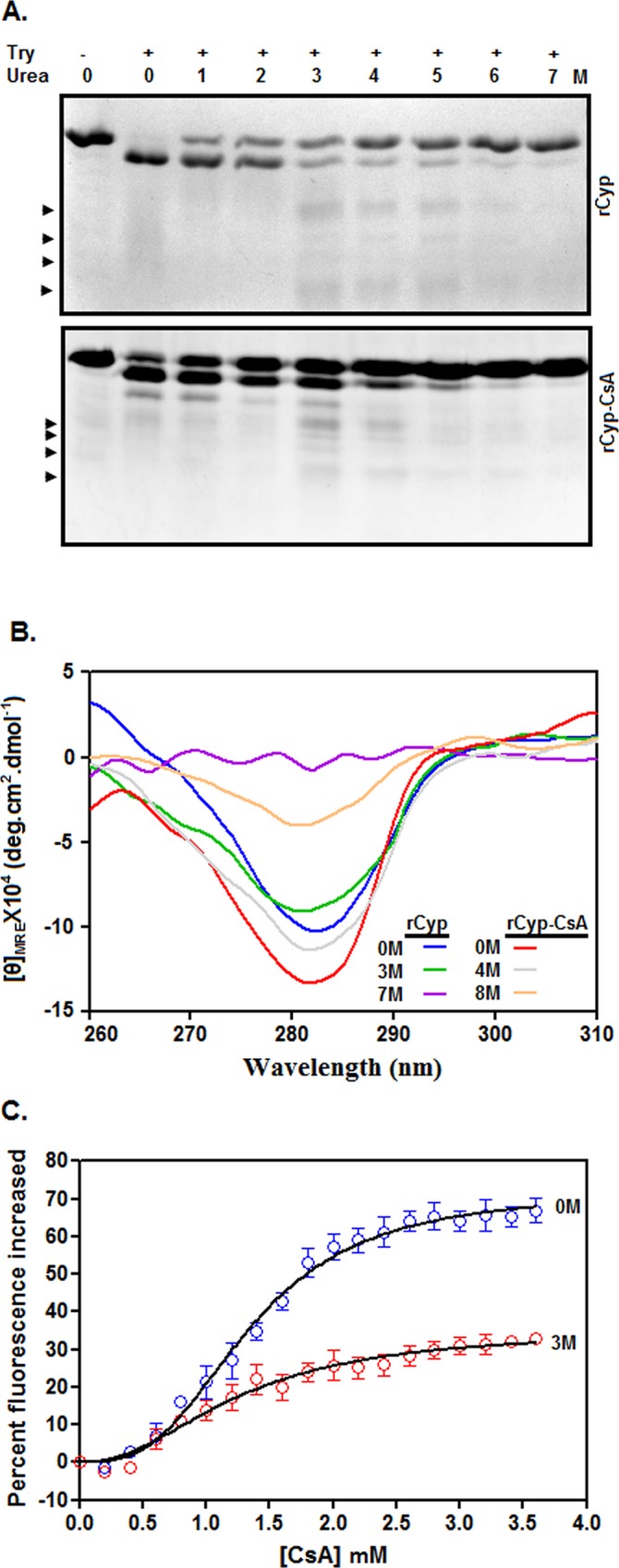
Properties of urea-made intermediates. (A) Analyses of the trypsin-generated fragments from rCyp and rCyp-CsA. Proteins were exposed to 0–7 M urea followed by their digestion with (+)/without (-) trypsin for 10 min at 25°C. All of the proteolytic fragments are resolved by SDS-13.5% PAGE. Arrowheads denote new protein fragments. (B) The near-UV CD spectra of rCyp and rCyp-CsA at the denoted concentrations of urea. The spectra were recorded using the same concentrations of proteins. (C) Drug binding assay. The curves show the change of Trp fluorescence intensity of 0 M and 3 M urea-exposed rCyp (2 μM) in the presence of 0–3.5 μM CsA.

To confirm the generation of unfolding intermediates, the urea-exposed rCyp and rCyp-CsA were separately digested with trypsin as stated [57]. The yielded proteolytic patterns of proteins at ~0–1 M urea look different from those in the presence ~2–6 M urea (Fig 6A). While new proteolytic fragments from rCyp appeared at ~3–6 M urea, those from rCyp-CsA are generated at ~3–4 M urea. The emergence of the additional proteolytic fragments might be due to the change of protein structure at the above urea concentrations. Thus, the data prove the production of unfolding intermediates from both proteins at moderately higher urea concentrations.

The ellipticity value of rCyp at 222 nm was reduced about 4% when we enhanced the urea concentration from 0 M to 3 M urea (S4A Fig), whereas, that of rCyp-CsA was dropped about 17% upon increasing the urea concentration from 0 M to 4 M urea. Therefore, both rCyp and rCyp-CsA intermediates are composed of sufficient extents of secondary structures.

The Trp fluorescence intensity of rCyp was decreased by ~40% upon augmenting the urea concentrations from 0 M to 3 M (S4B Fig). Conversely, there was a ~13% reduction of the Trp fluorescence intensity of rCyp-CsA when the urea concentration was enhanced from 0 M to 4 M. At the intermediate forming urea concentrations, the spectra of rCyp and rCyp-CsA are associated with the 4 nm and 2 nm red-shifted emission maxima, respectively. In sum, the tertiary structures of rCyp and rCyp-CsA intermediates may be partly different from those of the native forms of these proteins.

The ANS fluorescence intensities of rCyp at 3 M and rCyp-CsA at 4 M urea, unlike their far-UV CD and Trp fluorescence intensities, are more than 70% less in comparison with those of the related proteins at 0 M urea (S4C Fig). Therefore, the extent of the hydrophobic surface area in either intermediate is significantly less than that in the related native protein.

The near-UV CD values of rCyp at ~280–285 nm were slightly decreased when urea concentrations were augmented from 0 M to 3 M urea (Fig 6B). On the contrary, the near-UV CD values of rCyp-CsA at ~280–285 nm were marginally reduced upon raising the urea concentrations from 0 M to 4 M. Collectively, both intermediates retained sufficient extent of tertiary structures.

To verify whether rCyp intermediate is biologically active, we estimated the Trp fluorescence change of both 0 M and 3 M urea-equilibrated rCyp in the presence of different concentrations of CsA (Fig 6C). The yielded *K*_d_ values for the rCyp and CsA interaction at 0 M and 3 M urea are 1.34 ±0.05 μM and 1.25±0.13 μM, respectively. Further analysis reveals no significant change of *K*_d_ value (*p* = 0.23) upon changing the urea concentrations from 0 M to 3 M, indicating that rCyp intermediate did not lose any drug binding activity.

## Discussion

The present study has provided some seminal clues about the folding-unfolding mechanism and the domain structure of rCyp (Fig 1A), a chimeric SaCyp harboring 220 amino acid residues [42]. Our limited proteolysis (Fig 1) and the subsequent analyses (Table 1) have revealed that two rCyp ends carrying residues 1 to 22 and 218 to 220 are only susceptible to three proteolytic enzymes employed in the study. The rCyp region having residues 23 to 218 carries most of the cleavage sites of these enzymes (Fig 1A). The absence of digestion in the internal rCyp region indicates the formation of a domain by the residues 23 to 218. The residue 23 in rCyp corresponds to the C-terminal end residue of its polyhistidine tag, whereas its residue 218, equivalent to the residue 195 of SaCyp (Fig1A), is the C-terminal end residue of the domain. Thus, the single domain structure of SaCyp proposed before on the basis of computational studies [42] was confirmed by our proteolysis results. However, such single domain structure is not unprecedented as the cyclophilins those have masses nearly similar to that of SaCyp are also shown to carry single domain capable of binding both the substrate and inhibitor [2, 3, 11, 19].

The proteolysis of Val 218—Glu 219, and Glu 219—Glu 220 peptide bonds (Fig 1) indicates that the rCyp residues Val 218, Glu 219, and Glu 220, corresponding to SaCyp residues Val 195, Glu 196, and Glu 197, might be exposed to its surface. An examination of the model SaCyp structure [42] using Swiss PDB Viewer (spdv.vital-it.ch) reveals that four C-terminal end residues, Asp 194, Val 195, Glu 196 and Glu 197, are not involved in the formation of any secondary structure and more than 20% exposed to its surface. We have noted that the extreme C-terminal end of some SaCyp homologs [64–66], formed by two to six residues, are also not structured but adequately surface-exposed. The above data not only support our proteolysis data but also indicate that a short flexible region, made with two amino acid residues, is attached to the C-terminal end of SaCyp domain. Currently, little is known about the structural and functional significance of the above tail.

Our spectroscopic data have indicated that unfolding of rCyp or rCyp-CsA at 0-7/8 M urea proceeds via the synthesis of one stable intermediate (Fig 5). The unfolding pathway of either protein in the presence of urea was fully reversible though there was the production of an intermediate (Fig 4). The surface hydrophobicity, secondary structure, and the tertiary structure rCyp intermediate are not fully identical to those of rCyp-CsA intermediate (Fig 6 and S4 Fig). The surface hydrophobicity of the intermediates also does not match with those of native proteins. On the other hand, the intermediates, compared to the native proteins, only marginally lost their secondary or tertiary structure. Of the intermediates, the rCyp intermediate is formed at comparatively less concentration of urea (Fig 5). Collectively, the number and type of non-covalent interactions responsible for stabilization of a protein structure [67, 68] are possibly not identical in the two intermediates.

An earlier study indicated that the GdnCl-induced equilibrium unfolding of rCyp or rCyp-CsA proceeds by a three-state mechanism via the production of an intermediate [42]. Therefore, the unfolding mechanism of rCyp and rCyp-CsA in the presence of urea (Fig 5) matches with that of these proteins in the presence of GdnCl [42]. Despite the identical mechanism, the structural properties of the urea-made intermediates are not completely identical to those of the GdnCl-generated intermediates (Fig 6). The secondary structure [42] and the tertiary structure (S5A Fig) of the GdnCl-made rCyp intermediate, unlike those of the urea-produced rCyp intermediate (Fig 6B and S4 Fig), were severely affected. On the other hand, the secondary structure [42] and the tertiary structure of the GdnCl-made rCyp-CsA intermediate or the urea-created rCyp-CsA intermediate are very similar to those of native protein. Interestingly, all of the intermediates possess somewhat a reduced extent of hydrophobic surface area (S4C Fig and S5B Fig). Of the different types of protein folding-unfolding intermediates proposed previously [69–71], dry molten globules usually possess a native-like secondary structure and tertiary structure but have different side chain packing. All of the rCyp/rCyp-CsA intermediates, except GdnCl-made rCyp intermediate, therefore, could be dry molten globules as their structures closely resemble those of the corresponding native proteins. Currently, little is known about the status of side-chain packing in the above intermediates.

The unfolding mechanism of drug-bound/unbound rCyp shows some similarity and dissimilarity with those of drug-bound/unbound CPR3, LdCyp, and PpiA [40, 41, 43]. The latter proteins are single domain cyclophilins having 40–44% sequence identity with SaCyp. CPR3, encoded by yeast, was unfolded by means of the creation of two structurally different intermediates in the presence of urea [43]. Of the CPR3 intermediates, the intermediate formed at relatively less urea concentration has the characteristics of a molten globule [71]. Like rCyp, LdCyp, synthesized by *Leishmania donovani* [41], was unfolded by a three-state mechanism in the presence of GdnCl. The LdCyp intermediate, like the above CRP3 intermediate, also has the properties of a molten globule [71]. On the other hand, the urea-induced unfolding of PpiA (a mycobacterial cyclophilin) or its drug-bound form occurred via the formation of an intermediate [40]. Currently, little is known about the biological activities of CPR3, LdCyp, and PpiA intermediates. Conversely, our studies for the first time have indicated that the drug binding activities of the urea-made rCyp intermediate and native rCyp are nearly similar (Fig 6C). The GdnCl-made rCyp intermediate also retained about 25% of the total drug binding activity of native rCyp (S5 Fig).

The complete retention of drug binding activity in the rCyp intermediate (Fig 6C) implies no significant alteration of the three-dimensional structure of the cyclosporin A binding site in the presence of 3 M urea. Our previous studies showed that the putative cyclosporin A binding site in SaCyp is primarily located in the regions harboring residues ~Arg 59 to Phe 116 and ~Trp 152 to His 157 [42]. Therefore, the structural change noted in rCyp intermediate (Fig 2) possibly have occurred at regions carrying residues ~Ala 2 to His 58, ~Ile 117 to Pro 151, and ~Thr 158 to Glu 197. Besides Trp 152, SaCyp carries another Trp residue at position 136 [42]. An analysis of the model SaCyp structure [42] with Swiss PDB Viewer (spdv.vital-it.ch) indicates that the former Trp residue is relatively more exposed on the surface of SaCyp. As Trp 152 is conserved and indispensable for binding CsA [1], there might be little change of the structure around this residue in the rCyp intermediate. The altered Trp fluorescence intensity and emission maxima of the rCyp intermediate (Fig 2B and 2C), therefore, suggests a structural change around Trp 136. Additional studies are needed to prove the urea-induced structural alteration around Trp 136 with certainty.

Many promising CsA analogs with no immunosuppressive activity were discovered and suggested to be useful in treating various diseases [2, 16–18, 72]. As these analogs did not yield encouraging results in the clinical trials [10, 11], screening or synthesis of additional CsA analogs should be continued on a priority basis. An inhibitor can be easily screened against a drug target if the binding of the former increases the midpoint of unfolding transition (or the stability) of the latter [33–39]. Several chemical denaturation-based assay systems were reported to screen the drug molecules against various drug targets including PPIase enzymes [28, 34, 35, 37, 39]. Our present (Table 2) and previous [42] unfolding results demonstrated the significant increment of the stability of rCyp in the presence of CsA. The urea-induced unfolding of a mycobacterial cyclophilin also reported its stabilization by CsA [40]. Collectively, an unfolding-based assay system could be developed using SaCyp or rCyp for screening new CsA analogs in the future.

## Conclusions

Our investigations have provided invaluable clues about the basic structure and the folding-unfolding mechanism of SaCyp, an *S*. *aureus*-encoded cyclophilin involved in pathogenesis. We noted that rCyp, a recombinant SaCyp, is a single-domain protein with a short tail at its C-terminal end. Additionally, rCyp unfolds via the formation of an intermediate in the presence of urea. The rCyp intermediate has the native-protein like structure and also shows little loss of CsA binding activity. The unfolding of the CsA-bound rCyp also similarly occurred in the presence of urea. The stability data of rCyp seems to be applicable in the discovery of new CsA derivatives in the future.

## Supporting information

S1 Fig Analysis of the proteolytic fragments by MALDI-TOF mass spectroscopy. The fragments resulted from the digestion of rCyp with proteinase K (A), trypsin (B), and chymotrypsin (C) were processed (as mentioned in Materials and methods) followed by the recording of their spectra using MALDI-TOF equipment. The ‘m’ and ‘z’ indicate mass and charge number of ions, respectively.
(TIF)

S2 Fig Unfolding of proteins. Far UV CD (A and B), intrinsic Trp fluorescence (C and D), ANS fluorescence (E and F) of rCyp (A, C, and E) and rCyp-CsA (B, D, and F) in the presence of denoted concentrations of urea.
(TIF)

S3 Fig Proof of unfolding rCyp intermediate. (A) The fraction of unfolded rCyp molecules, calculated using the θ_222_ (Fig 2A) or Trp fluorescence intensity (Fig 2B) values and a standard equation [24], were plotted against 0–7 M urea. (B) Phase diagram shows the unfolding of rCyp at 0–7 M urea. *I*_320_ and *I*_365_ indicate the Trp fluorescence intensity values (extracted from S2C Fig) of rCyp at 320 nm and at 365 nm, respectively.
(TIF)

S4 Fig Structure of urea-made intermediates. The far-UV CD (A), intrinsic Trp fluorescence (B), and ANS fluorescence (C) spectra of rCyp and rCyp-CsA at the denoted concentrations of urea. The spectra at the indicated urea concentrations were collected from S2 Fig.
(TIF)

S5 Fig Characteristics of intermediates made by GdnCl. The near-UV CD (A) and the ANS fluorescence (B) spectra of rCyp and rCyp-CsA at the shown GdnCl concentrations. The spectra were recorded using equimolar concentrations of proteins. The rCyp and rCyp-CsA intermediates were formed at 1.5 M and 0.6 M GdnCl, respectively [42]. (C) CsA binding assay. The curve represents the alteration of Trp fluorescence intensity of 1.5 M GdnCl-treated rCyp (2 μM) in the presence of 0–3.5 μM CsA.
(TIF)
