# Supplementary figures and images for: A staphylococcal cyclophilin carries a single domain and unfolds via the formation of an intermediate that preserves cyclosporin A binding activity
Source: PLoS One. 2019 Mar 29;14(3):e0210771. doi: 10.1371/journal.pone.0210771 (PMC6440624; doi:10.1371/journal.pone.0210771)

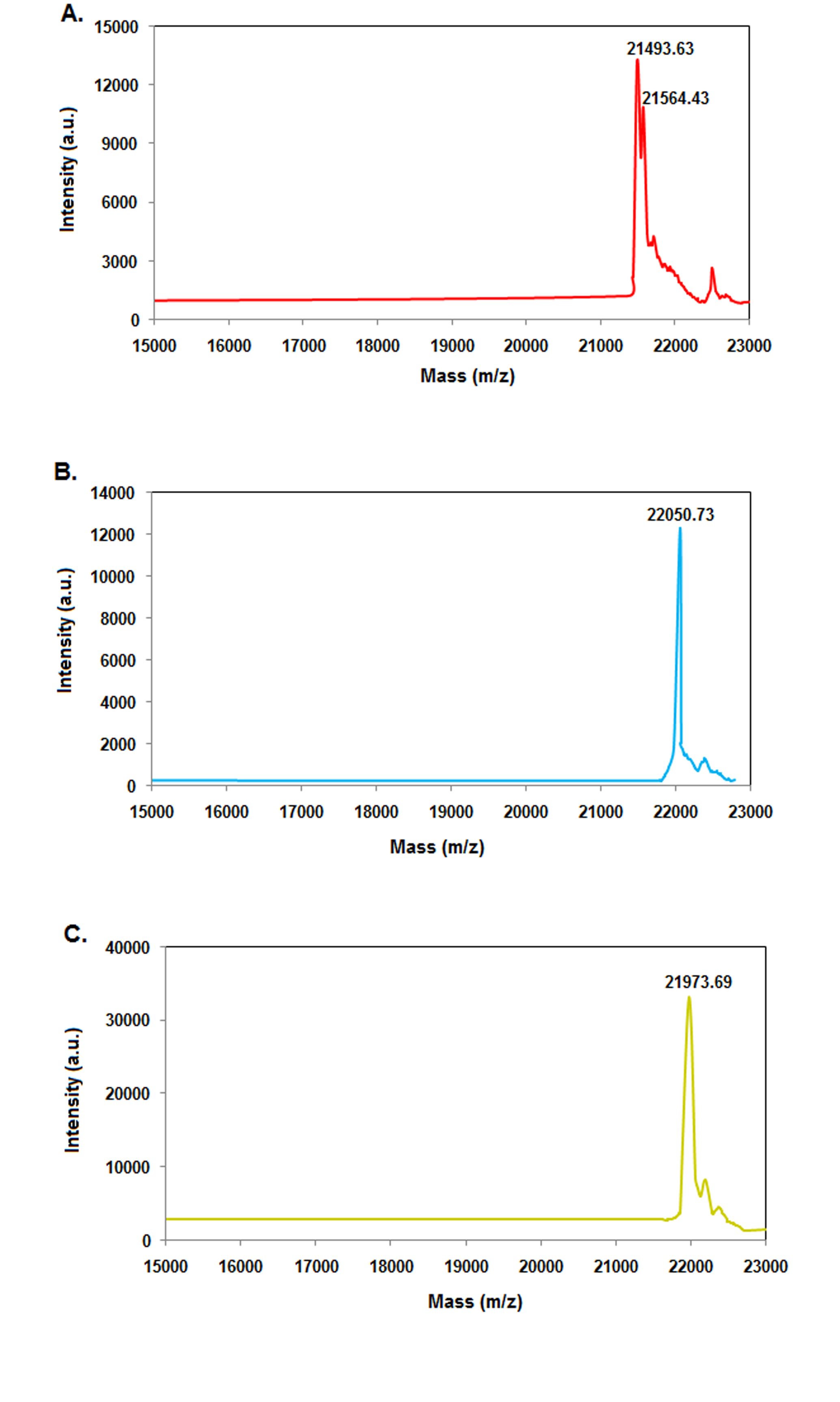

Supplement: S1 Fig — The fragments resulted from the digestion of rCyp with proteinase K (A), trypsin (B), and chymotrypsin (C) were processed (as mentioned in Materials and methods) followed by the recording of their spectra using MALDI-TOF equipment. The ‘m’ and ‘z’ indicate mass and charge number of ions, respectively. (TIF) [file pone.0210771.s001.tif]

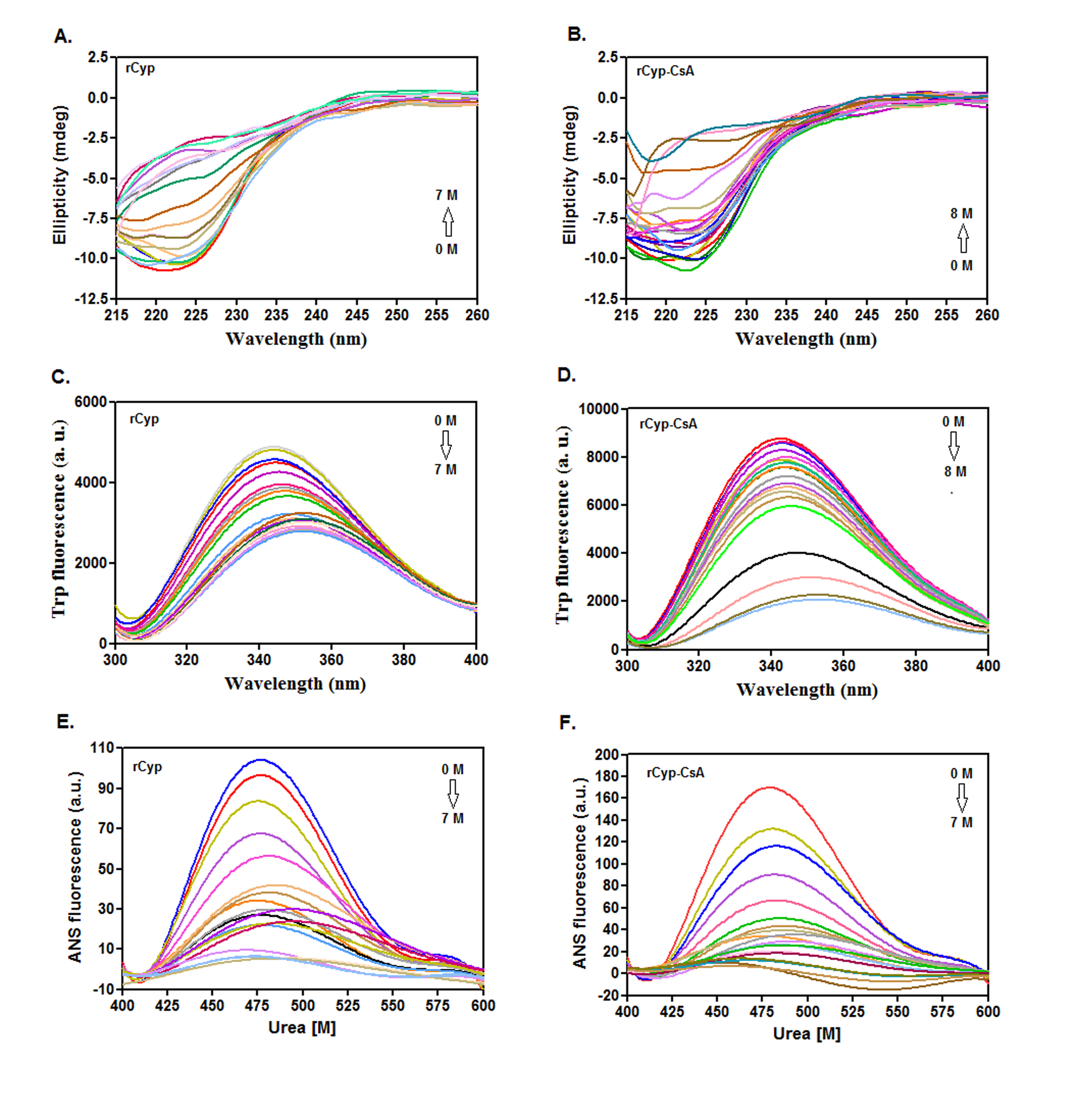

Supplement: S2 Fig — Far UV CD (A and B), intrinsic Trp fluorescence (C and D), ANS fluorescence (E and F) of rCyp (A, C, and E) and rCyp-CsA (B, D, and F) in the presence of denoted concentrations of urea. (TIF) [file pone.0210771.s002.tif]

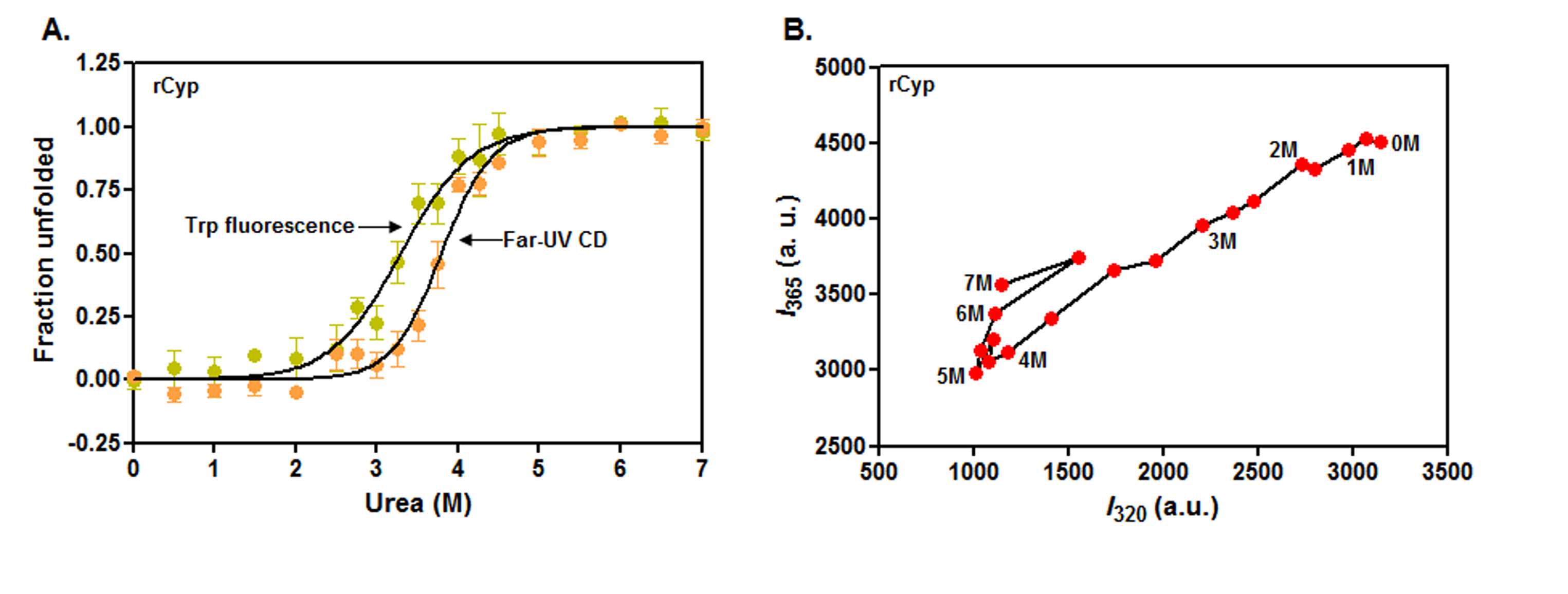

Supplement: S3 Fig — (A) The fraction of unfolded rCyp molecules, calculated using the θ222 (Fig 2A) or Trp fluorescence intensity (Fig 2B) values and a standard equation [24], were plotted against 0–7 M urea. (B) Phase diagram shows the unfolding of rCyp at 0–7 M urea. I320 and I365 indicate the Trp fluorescence intensity values (extracted from S2C Fig) of rCyp at 320 nm and at 365 nm, respectively. (TIF) [file pone.0210771.s003.tif]

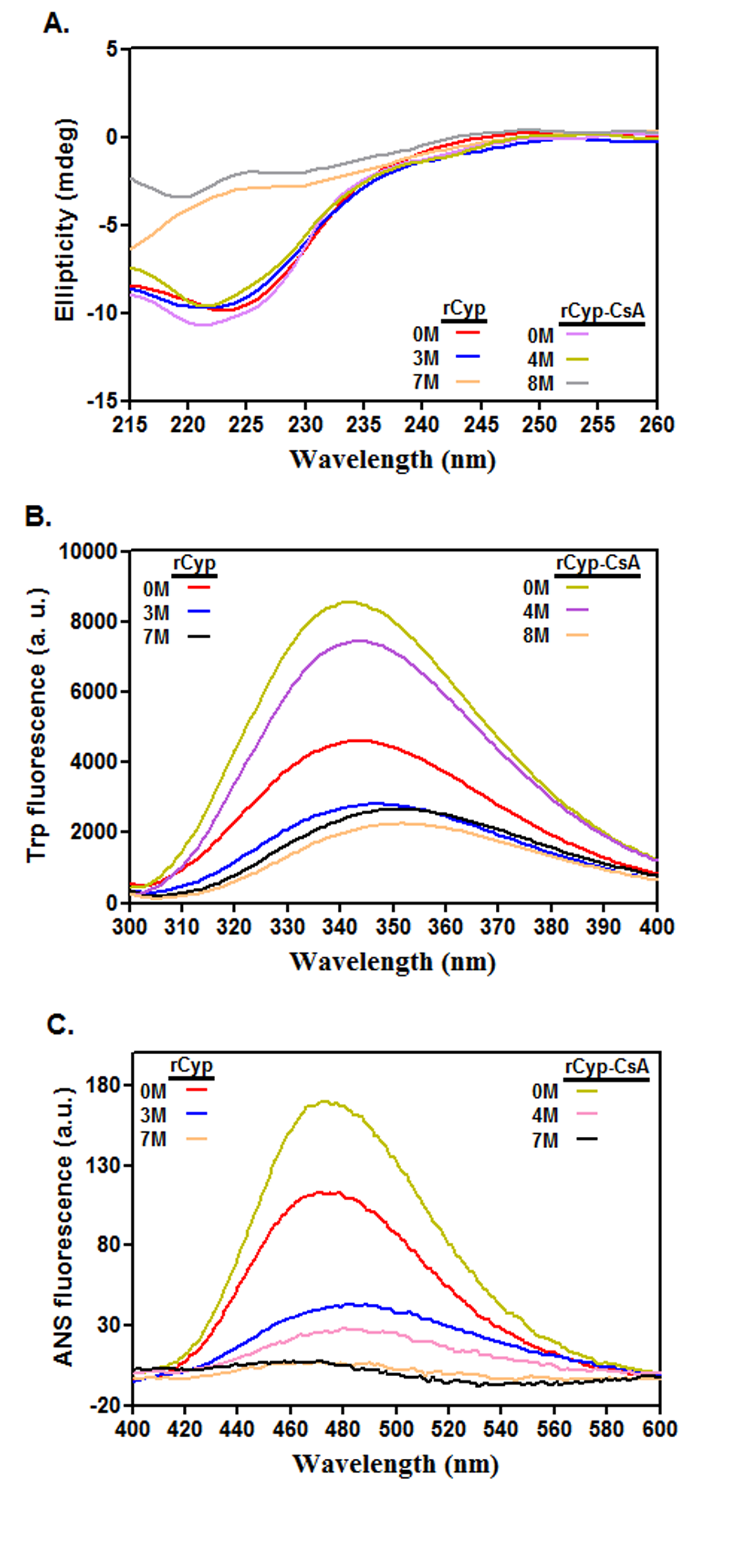

Supplement: S4 Fig — The far-UV CD (A), intrinsic Trp fluorescence (B), and ANS fluorescence (C) spectra of rCyp and rCyp-CsA at the denoted concentrations of urea. The spectra at the indicated urea concentrations were collected from S2 Fig. (TIF) [file pone.0210771.s004.tif]

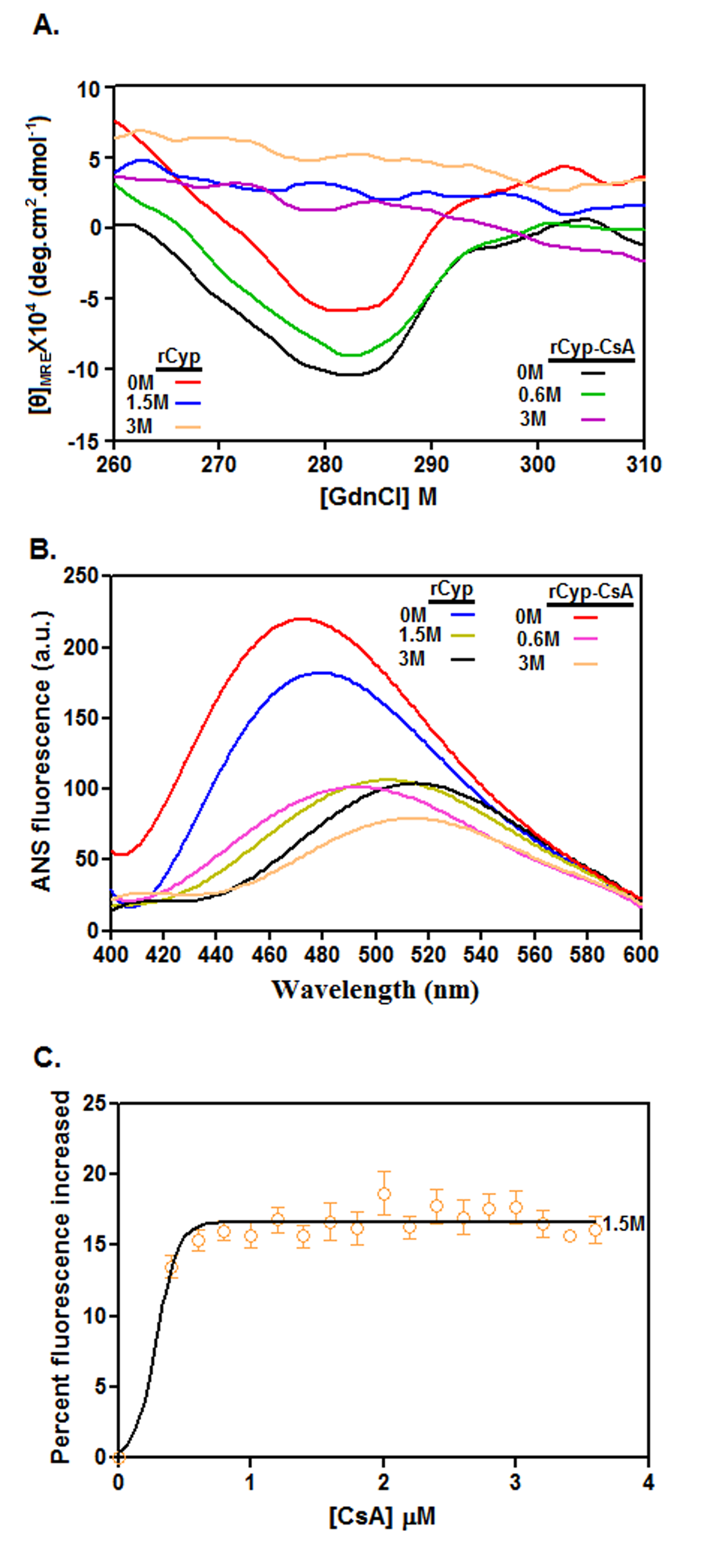

Supplement: S5 Fig — The near-UV CD (A) and the ANS fluorescence (B) spectra of rCyp and rCyp-CsA at the shown GdnCl concentrations. The spectra were recorded using equimolar concentrations of proteins. The rCyp and rCyp-CsA intermediates were formed at 1.5 M and 0.6 M GdnCl, respectively [42]. (C) CsA binding assay. The curve represents the alteration of Trp fluorescence intensity of 1.5 M GdnCl-treated rCyp (2 μM) in the presence of 0–3.5 μM CsA. (TIF) [file pone.0210771.s005.tif]
